# Efficacy of Red Cell Salvage Systems in Open Acetabular Surgery

**DOI:** 10.1155/2022/8276065

**Published:** 2022-06-02

**Authors:** S. MacDonald, C. Byrd, E. Barlow, V. K. Nahar, J. Martin, D. Krenk

**Affiliations:** ^1^East Tennessee State University, Quillen College of Medicine, Orthopedic Residency, Johnson City, TN, USA; ^2^University of Mississippi Medical Center, Jackson, MS, USA

## Abstract

Over the past 50 years, treatment of displaced acetabular fractures has moved away from conservative treatment with bedrest to operative intervention to achieve anatomic reduction, stable fixation, and allow early range of motion of the hip. However, operative fixation is not without complications. Internal fixation of traumatic acetabular fractures has been coupled with large volume of blood loss both at the time of injury and surgery. This often results in the need for allogenic blood products, which has been linked to increase morbidity (Vamvakas and Blajchman, 2009). In an attempt to avoid the risk associated with allogenic blood transfusion numerous techniques and methods have been devised. Red blood cell salvage (CS) is an intraoperative blood salvage tool where blood is harvested from the operative field. It is washed to remove the plasma, white blood cells, and platelets. The red cells are resuspended in a crystalloid solution. If the hematocrit of the resuspended red blood cells is sufficient, it is transfused to the patient intravenously. The benefits of CS in major spine surgery, bilateral knee replacement, and revision hip surgery are well established (Goulet et al. 1989, Gee et al. 2011, Canan et al. 2013). However, literature reviewing the use of cell saver in orthopedic trauma surgery, specifically acetabular surgery is limited. Our institute performed a retrospective review of 63 consecutive operative acetabular fractures at a level one trauma center. Our study revealed that patients with blood loss of less than 400 mL were 13 times less likely to receive autologous blood, and patients with hemoglobin less than 10.5 were 5 times less likely to receive autologous transfusion (*p* < 0.05). We also found that no patients with a hemoglobin level less than 10.5 and EBL less than 400 mL received autologous blood return. Autologous blood transfusion had no effect on volume or rate of allogenic blood transfusion. We believed that if a patient's preoperative hemoglobin is less than 10.5 or expected blood loss is less than 400 mL, then CS should have a very limited role, if any, in the preoperative blood conservation strategy. We found ASA greater than 2, BMI greater than 24 and associated fracture type to be a risk factor for high blood loss.

## 1. Introduction

Historically, acetabular fractures were often treated with nonoperative management and bedrest. Judet et al. brought about a change in this paradigm with their published report on acetabular fracture patterns and surgical anatomy in 1964 [1]. The body of literature regarding treatment of acetabular fractures was enhanced when Tornetta published his 2001 article on indications for operative and nonoperative management of acetabular fractures [2]. The outcomes of open reduction internal fixation of acetabular fractures have overall been reported as good [3]. Thus, the trend toward operative treatment to achieve anatomic reduction, ridged internal fixation, and to allow early mobilization became the preferred treatment of most displaced acetabular fractures. However, operative fixation is not without its own complications. Surgical treatment of acetabular fractures has been coupled with large blood loss, both at the time of injury and surgery. This often results in the need for blood products, which have been linked to increased morbidity and mortality [4, 5].

In an attempt to avoid the risk associated with allogenic blood transfusion, numerous techniques and methods have been devised. These include preoperative autologous blood donation, blood dilution, intraoperative hypotension, pharmaceutical blood substitutes, tranexamic acid, and intraoperative red cell salvage (CS). Predonated blood is not an option in trauma patients. Negative aspects of these treatments include cost, risk of end organ damage with prolonged hypotension, and increased mortality at one year [6, 7]. In opposition, intraoperative CS has not been associated with adverse clinical outcomes [6].

CS is an intraoperative blood salvage tool where blood is harvested from the operative field. Then, the blood is washed to remove the plasma, white blood cells, and platelets. The red cells are resuspended in a crystalloid solution, and if the hematocrit of the resuspended red blood cells is sufficient, it is transfused to the patient. The average hematocrit of this autologous blood has been reported to be 55% [8, 9].

The benefits of CS in major spine surgery, bilateral knee replacement, and revision hip surgery are well established [10–12]. However, literature reviewing the use of cell saver in orthopedic trauma surgery, specifically acetabular surgery is limited. The aim of this retrospective study is to evaluate the effectiveness of CS in decreasing allogenic blood transfusion in acetabular surgery, contribute to the body of literature regarding cell saver use in acetabular fractures, and to identify risk factors for large intraoperative blood loss.

## 2. Methods

Following Institutional Review Board's approval, a retrospective review was performed at a level 1 trauma center of all operative acetabular fractures performed during a 5-year period. CS with the LivaNova XTRA ATS system was routinely used on all acetabular reconstructions. All cases were performed by a single fellowship trained pelvic and acetabular surgeon. There was a total of 93 operative acetabular fractures in 93 patients. Patients excluded from the study included those treated percutaneously (*n*-28) or incomplete medical record (*n*-2). Medical records were used to obtain demographics, American Society of Anesthesiologist Scores (ASA), fracture classification, approach, laboratory data, volume of autologous blood transfused, rate of autologous blood transfusions, preoperative allogenic blood transfusion, intraoperative allogenic blood transfusion, postoperative allogenic blood transfusion, and days to surgery (DTS). We compared those who received autologous blood transfusion to those who did not in an attempt to identify those who would benefit the most from it use. Subgroup analysis was performed in an attempt to find predictors of high EBL, autologous transfusion, and allogenic transfusion.

No protocol was used in determining the need for allogenic blood transfusion. Perioperative and postoperative allogenic transfusion was initiated by the anesthetist or general surgery trauma team based on clinical and physiological parameters. Using the Letournel classification, acetabular fractures were classified into elementary fracture (EF) and associated fractures (AF) [1]. Fractures were classified by the operative surgeon. Time to surgery was rounded to the nearest day. EBL was determined by the operative surgeon and anesthesiology team. The amount of autologous blood transfused was recorded in the operative report and perfusionist notes. CS was used in all open operative cases per the operating surgeon's protocol. For all cases, blood was collected from incision to skin closure. Based on the blood volume and hematocrit of the collected blood, the decision was made by the perfusionist whether the volume and hematocrit of the collected blood was sufficient for autologous transfusion. If the CS collected blood volume and hematocrit was sufficient, a centrifuge was used to separate the red blood cells and plasma components, the red cells were then washed and resuspended in a crystalloid solution and transfused to the patient.

Data measurements were recorded in means unless specified. For the data recorded in the interval scale, a t-test was used to compare between groups. Chi square test was used in the evaluation of nominal data. Subset analysis was performed based on fracture classification, BMI, EBL, preoperative hemoglobin, ASA score, and time to surgery.

## 3. Results

Ninety-three patients who underwent internal fixation of an acetabular fracture were identified. Twenty-eight were excluded because they underwent only percutaneous procedure, and two were excluded due to inadequate documentations in the medical record. There were 16 women and 47 men. The mean age was 45 (range 20–79), mean time to surgery was 4 days (range 1–12), mean BMI was 28.7 (range 16.3–51.4), mean preoperative hemoglobin was 11.07 (range 8.3–15.4), mean postoperative hemoglobin was 10.4 (range 8–14.5), and mean EBL 256 mL (range 150–600). Eight (12.7%) received autologous blood transfusions, whereas 55 (87.3) were unable to received autologous blood transfusion because the hematocrit failed to meet the minimum of 40% required for autologous transfusion. Twenty-nine (46%) had elementary fractures, where 34 (54%) had associated fracture patterns. Four (6%) underwent Ilioinguinal (IL) approach, whereas 59 (94%) were treated with a Kocher-Langenbeck (KL) approach. Seven (11%) of those treated with the KL approach also had anterior percutaneous fixation. Three percent had an ASA of 1, 41% had and ASA of 2, 52% had an ASA of 3, and 3% had an ASA of 4 (Table 1).

Patients who received autologous blood transfusion were compared to those who did not receive autologous blood transfusion. Significant differences in BMI and EBL were noted. Those who received autologous blood transfusion had a higher BMI (33.7 vs 27.9, *P* < 0.05) and greater blood loss (459 mL vs 227 mL, *P* < 0.05). There was no difference in age, gender, ASA score, preoperative hemoglobin, postoperative hemoglobin, allogenic transfusion volume, rate of allogenic blood transfusion, surgical approach, and fracture type (Table 2).

Analysis was performed to determine if gender, BMI, ASA, fracture pattern (elementary vs associated), preoperative hemoglobin, and surgical approach were predictors of intraoperative blood loss. Increased intraoperative blood loss was associated with the ASA score >2 (304 mL vs 228 mL, *P* < 0.05), BMI greater than 24 (275 mL vs 191 mL, *P* < 0.05), and associated fracture type (289 mL vs 219 mL, *P* < 0.05) (Table 3).

Preoperative hemoglobin of less than 10.5 mg/dl was associated with older age, ASA >2, higher volume of intraoperative blood transfused, as well as higher rate of preoperative, intraoperative, and postoperative allogenic blood transfusions. The rate of autogenous blood transfusion was also significantly less when preoperative hemoglobin was less than 10.5 mg/dl (Table 4).

Patients with EBL >400 ml were more likely to have an elevated BMI and received higher volumes of blood when autologous transfusion was provided. EBL>400 ml was associated with a higher rate of postoperative allogenic blood transfusion, IL surgical approach, and autologous blood transfusion (Table 5).

Patients with preoperative hemoglobin less than 10.5 mg/dl were 5 times less likely to receive autologous blood transfusion compared to patients with hemoglobin >10.5 mg/dl, and (1/26 (4%) vs 7/37 (19%), *P* < 0.05). Autologous transfusion was also 20 times more likely when blood loss exceeded 400 mL (6/13 (46%) vs 2/50 (4%), *P* < 0.05) (Table 6).

## 4. Discussion

At our institution, CS is used on all patients undergoing open operative acetabular fracture repair. Sixty-three consecutive open operative acetabular fractures repairs were assessed. This investigation was designed to determine if routine use of CS in acetabular fracture repair is efficacious in reducing the rate of allogenic blood transfusion and to identify predictors of large blood loss. The volume and or hematocrit in the harvested blood was sufficient enough for return in only 8/63 (13%) patients. Previously studies have reported rate of CS return ranging from 20%–81% (7, 13). The only significant difference between the CS return group and no CS return group was BMI and EBL. No statistical difference was noted in allogenic transfusion rate between these two groups; this is consistent with previous reports [13].

We found that the patients who underwent the IL approach had an average blood loss of 425 ml compared to 245 ml in the KL approach. Only 25% (1/4) of the IL approach and 12% (7/59) of the KL approach received CS return and 50% of the IL approach and 15% of the KL approach received a postoperative allogenic blood transfusion. The patients who received a postoperative allogenic blood transfusion had a preoperative hemoglobin average of 10.1 mg/dl. Eight of the eleven patients (73%) who received a postoperative blood transfusion had a preoperative hemoglobin below 10.4 mg/dl. We believe a preoperative hemoglobin level below 10.4 mg/dl is a stronger indicator of patients whom may need an allogenic blood transfusion postoperatively regardless of the approach.

We found that patients with an ASA score greater than 2, BMI greater than 24, and associated fractures had statistically significant higher blood loss. Like Firoozabadi et al., we suspect that low average blood volume loss in our patients resulted in a low rate of autogenic blood transfusion [14]. We believe the ability of cell saver to produce blood sufficient for return to be dependent on both the quantity and quality of the blood harvested from the field. Our studies have shown that patients with blood loss in excess of 400 mL were 20 times more likely to receive autologous blood; this is consistent with previous finding [14]. What has not been demonstrated prior to this study is that the quality of the blood harvested from the field also contributes to the success of CS to provide sufficient blood for autologous transfusion.

In our study, patients with preoperative hemoglobin less than 10.5 mg/dl were 5 times less likely to receive autologous transfusion. We also found that no patients with a preoperative hemoglobin less than 10.5 mg/dl and EBL less than 400 mL received autologous blood return. We believed that preoperative hemoglobin less than 10.5 mg/dl makes it difficult to achieve the minimum hematocrit of 40% for reinfusion of collected red cells. Because of this, if a patient's preoperative hemoglobin is less than 10.5 mg/dl or expected blood loss is less than 400 mL, we believe CS should have a very limited role if any in the preoperative blood strategy due to the low likelihood of obtaining quality red cells for reinfusion.

Previous studies have reported an anterior approach and male gender to be associated with high EBL [14]. In our study, we found that BMI greater than 24, ASA greater than 2, and associated fractures to be associated with higher EBL.

The limitations of this study are that it is retrospective and there was no protocol to indicate when a patient would receive allogenic blood transfusion. The decision to provide allogenic transfusion intraoperatively was made by the anesthesiologist and the operative surgeon. Postoperatively, the decision for transfusion was made by the general surgery trauma team, as they were the admitting service. The reason for transfusion was not always documented and therefore not available for review. A large number of patients had concomitant injuries and confounding blood transfusion requirements.

In summary, the utility of CS may not be warranted for routine use in acetabular surgery. There was no demonstrable reduction in intraoperative or postoperative allogenic blood transfusion volume or rate. We found that low blood loss and low preoperative hemoglobin levels made it difficult for CS to meet the minimum hematocrit in the salvaged blood for autologous transfusion. We were unable to define a group that would benefit from the routine use of CS. However, we did find significantly higher blood loss in patients with an ASA greater than 2, BMI greater than 24, and associated fracture types. We found CS use to be impractical during acetabular surgery when a patient's hemoglobin is less than 10.5 mg/dl and blood loss less than 400 mL is expected.

## Figures and Tables

**Table 1:** Patient data including age, gender, BMI, ASA, pre- and postophemoglobin in mg/dl, average EBL, number of autologous transfusions, average units of autologous blood transfused, average number of units pre- and postallogenic transfusions, fracture patterns, and surgical approach. IL = Ilioinguinal, KL = Kocher-Langenbeck, ASA = American Society of Anesthesiology Score, BMI = body mass index, EBL = estimated blood loss, EF–elementary fracture pattern, AF = associated fracture pattern.

Average age	45
Females	16
Males	47
Average BMI	28.7
Average ASA	2.44
ASA 1	3%
ASA 2	41%
ASA 3	52%
ASA 4	3%
Average preoperative hemoglobin	11.1 mg/dl
Postoperative hemoglobin	10.4 mg/dl
Average EBL	256 ml
#Of patients receiving autologous blood transfusion	8 (13%)
Average preoperative allogenic blood transfusion (units)	0.48
Average intraoperative allogenic blood transfusion (units)	0.37
Average postoperative allogenic blood transfusion (units)	0.44
Elementary fractures-EF	29 (46%)
Associated fractures-AF	34 (54%)
IL approach	4 (6%)
KL approach	59 (94%)

**Table 2:** Comparison of patients who received autologous blood transfusion to those who did not. Elevated body mass index and higher estimated blood loss were significantly more likely to receive an autologous blood transfusion. There was no association between volume or rate of allogenic blood transfused preoperatively, intraoperatively, or postoperatively in patients who received an autologous transfusion compared to patients who did not. There was no association between surgical approach, gender, or fracture pattern and those receiving an autologous transfusion. IL = Ilioinguinal, KL = Kocher-Langenbeck, ASA = American Society of Anesthesiology Score, BMI = body mass index, EBL = estimated blood loss, EF–elementary fracture pattern, AF = associated fracture pattern.

	No autologous transfusion	Autologous transfusion	*P* value
Average age	45	24	0.63
Average BMI	27.9	33.7	<0.01
EBL (ml)	227	459	<0.01
ASA <2—ASA >2	31/55–24/55	4/8–4/8	0.74
Preoperative hemoglobin (mg/dl)	10.9	11.9	0.16
Days to surgery	4.3	4.0	0.57
Mean number of preoperative allogenic blood transfusion in units	0.5	0.25	0.57
Mean number of intraoperative allogenic blood transfusion in units	0.4	0.13	0.33
Mean number of postoperative allogenic blood transfusion in units	0.45	0.36	0.87
Rate of preoperative allogenic blood transfusion	11/55	1/8	0.47
Rate of intraoperative allogenic blood transfusion	13/55	1/8	0.48
Rate of postoperative allogenic blood transfusion	9/55	2/8	0.54
IL vs KL approach	3/55–52/55	1/8–7/8	0.45
Females - males	13/55–42/55	3/8–5/8	0.40
EF—AF	26/55–29/55	3/8–5/8	0.60

**Table 3:** Average EBL was analyzed for associations with ASA <2 vs ASA >2, BMI <24 vs BMI >24, elementary or associated fracture pattern, surgical approach, preoperative hemoglobin <10.5 vs >10.5 mg/dl, and gender. Patients with an ASA >2, BMI >24, and associated fracture patterns were associated with increased intraoperative blood loss. Surgical approach, postoperative hemoglobin, nor gender was associated with increased intraoperative blood loss.  = Ilioinguinal, KL = Kocher-Langenbeck, ASA = American Society of Anesthesiology Score, BMI = body mass index, EBL = estimated blood loss, EF–elementary fracture pattern, AF = associated fracture pattern.

	EBL	EBL	*P* value
ASA <2 vs ASA >2	228	304	0.03
BMI <24 vs BMI >24	191	275	<0.01
EF vs AF	219	288	0.04
IL vs KL approach	245	425	0.16
Preoperative hemoglobin <10.5 vs >10.5 mg/dl	242	267	0.44
Female vs male	290	245	0.45

**Table 4:** Preoperative hemoglobin <10.5 mg/dl was associated with older age, ASA >2, and higher volume of blood transfused. Risk of preop, intraop, and postop allogenic blood transfusion was higher in patients with preoperative hemoglobin less than 10.5 mg/dl. Rate of autologous blood transfusion was less in patients with preoperative hemoglobin less than 10.5 mg/dl. IL = Ilioinguinal, KL = Kocher-Langenbeck, ASA = American Society of Anesthesiology Score, BMI = body mass index, EBL = estimated blood loss, EF–elementary fracture pattern, AF = associated fracture pattern.

	Preoperative hemoglobin <10.5	Preoperative hemoglobin >10.5	*P* value
Average age	53	40	<0.01
Average BMI	27.3	29.7	0.13
ASA <2	10/26 (38%)	25/37 (68%)	0.02
ASA >2	16/26 (62%)	12/37 (32%)	
Days to surgery	4.31	4.32	0.98
Mean volume of autologous blood transfused (ml)	250	212	0.43
Mean EBL	242	267	0.44
Mean number of preoperative allogenic blood transfusion in units	0.77	0.27	0.11
Mean number of intraoperative allogenic blood transfusion in units	0.73	0.11	<0.01
Mean number of postoperative allogenic blood transfusion in units	0.88	0.14	0.06
Rate of preoperative allogenic blood transfusion	8/26	3/37	0.02
Rate of intraoperative allogenic blood transfusion	11/26	3/37	<0.01
Rate of postoperative allogenic blood transfusion	8/26	3/37	0.02
IL vs KL approach	0/26–26/26	4/37–33/37	0.08
Females—males	7/26–19/26	9/37–28/37	0.20
EF—AF	11/26–15/26	17/37–20/37	0.13
Rate of autologous blood transfusion	1/26	7/37	0.02

**Table 5:** Patients with EBL >400 ml were more likely to have an elevated BMI and received higher volumes of blood when autologous transfusion was provided. The rate of requiring a postoperative allogenic transfusion was higher when EBL >400 ml. The rate of having EBL >400 ml was higher in fractures requiring treatment with an anterior surgical approach. The rate of receiving an autologous transfusion was higher when EBL >400 ml. IL = Ilioinguinal, KL = Kocher-Langenbeck, ASA = American Society of Anesthesiology Score, BMI = body mass index, EBL = estimated blood loss, EF–elementary fracture pattern, AF = associated fracture pattern.

	EBL <400	EBL >400	*P* value
Average age	45	45	0.93
Average BMI	27.6	33.2	0.02
ASA <2—ASA >2	20/50–30/50	8/13–5/13	0.16
Preoperative hemoglobin (mg/dl)	11.0	11.2	0.73
Days to surgery	4.16	5.08	0.29
Mean volume of autologous blood transfused (ml)	125	247.5	<0.01
Mean number of preoperative allogenic blood transfusion in units	0.5	0.38	0.68
Mean number of intraoperative allogenic blood transfusion in units	0.38	0.31	0.73
Mean number of postoperative allogenic blood transfusion in units	0.24	1.23	0.18
Rate of preoperative allogenic blood transfusion	9/50	3/13	0.67
Rate of intraoperative allogenic blood transfusion	11/50	3/13	0.93
Rate of postoperative allogenic blood transfusion	6/50	5/13	0.03
IL vs KL approach	1/50–49/50	3/13–10/13	<0.01
Females—males	12/50–38/50	4/13–9/13	0.62
EF—AF	23/50–27/50	4/13–9/13	0.32
Rate of autologous blood transfusion	1/50	7/13	<0.01

**Table 6:** Patients with preoperative hemoglobin less than 10.5 mg/dl were 5 times less likely to receive autologous blood transfusion compared to patients with hemoglobin >10.5 mg/dl. Autologous transfusion was also 20 times more likely when blood loss exceeded 400 ml.

	+ Autologous transfusion	− autologous transfusion	Odds ratio
Hgb <10.5 mg/dl	1	25	0.17
Hgb >10.5 mg/dl	7	30	

EBL >400 ml	6	7	20.83
EBL <400 ml	2	48	

## Data Availability

The data are available in the orthopedic surgery residency at East Tennessee State University, Quillen College of Medicine and the Ballad Health IRB. Requests for data can be sent to bhresearchstartup@balladhealth.org.
